# Audit of Anticholinergic Burden (ACB) in Intellectual Disability Psychiatry Patients

**DOI:** 10.1192/bjo.2026.11744

**Published:** 2026-06-30

**Authors:** Laxmiram Bethina, Dipti Patil, Ibrahim Younes, Elabbas Wala, Wajiha zia

**Affiliations:** Pennine Care NHS Foundation Trust, Greater Manchester, United Kingdom

## Abstract

**Aims:**

The primary aim was to evaluate the anticholinergic burden (ACB) among adult patients in community intellectual disability (ID) psychiatry services. The objectives were to quantify ACB using the ACB Scale, assess adherence to best practices for medication review and side-effect monitoring, and identify variations in care to inform targeted quality improvement.

**Methods:**

A retrospective audit of 146 randomly selected patient records was conducted across five community ID teams within PCNFT. Data was collected between March and May 2025. Key measures included the documentation of medication indication, evidence of MDT review, cognitive monitoring, and the recording of specific anticholinergic side effects.

**Results:**

The audit revealed marked inconsistencies in care. While two boroughs achieved 100% compliance for documenting medication indications, others were as low as 7.7%. Cognition was monitored in only 44.5% of patients, the use of validated side-effect monitoring tools was quite low (e.g., LUNSERS score documented in 0.7% of cases). The documentation of anticholinergic side effects was poor, being formally mentioned in just 7.5% of records, despite 15.1% of the cohort having documented hallucinations.

**Conclusion:**

This audit provides clear evidence of deficiencies in the monitoring and management of anticholinergic burden. The findings confirm that ACB and its associated symptoms are not being monitored routinely. This reinforces the urgent need for a targeted quality improvement strategy, supported by effective resource allocation.

